# Review of the Preparation and Application of Porous Materials for Typical Coal-Based Solid Waste

**DOI:** 10.3390/ma16155434

**Published:** 2023-08-03

**Authors:** Jinsong Du, Aiyuan Ma, Xingan Wang, Xuemei Zheng

**Affiliations:** 1 College of Environmental and Chemical Engineering, Dalian University, Dalian 116622, China; 13733025795@163.com; 2 School of Chemistry and Materials Engineering, Liupanshui Normol University, Liupanshu 553004, China; zxm_lpssy19@163.com

**Keywords:** coal gangue, fly ash, energy and environmental protection, Si-based porous material, resource utilization

## Abstract

The discharge and accumulation of coal-based solid waste have caused great harm to the ecological environment recently. Coal-based solid wastes, such as coal gangue and fly ash, are rich in valuable components, such as rare earth elements (REY), silicon dioxide, alkali metal oxides, and transition metal oxides, which can be used to synthesize various functional Si-based porous materials. This article systematically summarizes the physicochemical characteristics and general processing methods of coal gangue and fly ash and reviews the progress in the application of porous materials prepared from these two solid wastes in the fields of energy and environmental protection, including the following: the adsorption treatment of heavy metal ions, ionic dyes, and organic pollutants in wastewater; the adsorption treatment of CO_2_, SO_2_, NO_x_, and volatile organic compounds in waste gas; the energy regeneration of existing resources, such as waste plastics, biomass, H_2_, and CO; and the preparation of Li–Si batteries. Combining the composition, structure, and action mechanism of various solid-waste-based porous materials, this article points out their strengths and weaknesses in the above applications. Furthermore, ideas for improvements in the applications, performance improvement methods, and energy consumption reduction processes of typical solid-waste-based porous materials are presented in this article. These works will deepen our understanding of the application of solid-waste-based porous materials in wastewater treatment, waste gas treatment, energy regeneration, and other aspects, as well as providing assistance for the integration of new technologies into solid-waste-based porous material preparation industries, and providing new ideas for reducing and reusing typical Chinese solid waste resources.

## 1. Introduction

**Figure 1:**
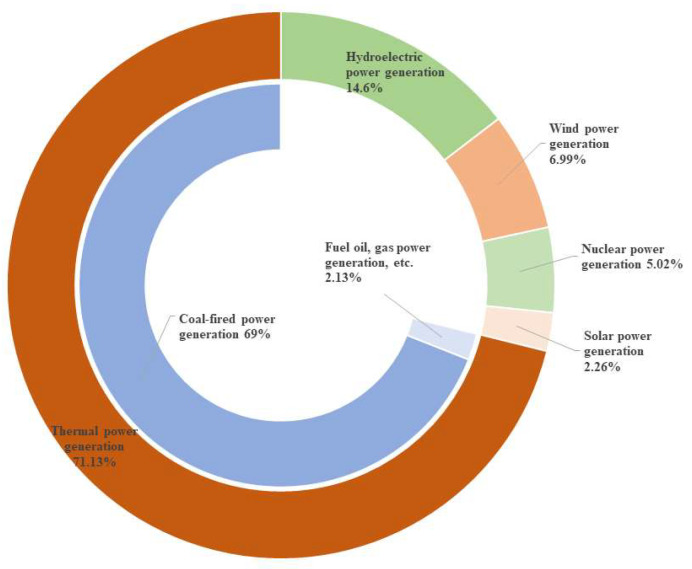
Composition of China’s electricity structure in 2021.

China is the world’s largest producer of coal and electricity. As of 2021, China’s power energy structure was still dominated by thermal power generation, with coal-fired power generation as its main component (Figure 1). The large output of coal and the large-scale application of coal-fired power result in a large amount of coal-based solid waste generation in China every year. According to the statistics, the annual output of coal gangue and fly ash in China currently exceeds 700 million tons. The discharge and accumulation of a large amount of coal-based solid waste have caused great harm to the ecological environment [1].

Over the past few decades, multiple countries have dedicated their efforts toward the utilization of coal-based solid waste, applying it in various fields, such as power generation, agriculture, construction materials, and others [2,3,4]. Recently, to achieve applications of coal-based solid waste with greater added value, numerous researchers have conducted new explorations in the field of functional materials. Several studies indicate that coal-based solid waste contains abundant silicon oxide and aluminum oxide, as well as valuable components, including rare earth elements, alkali metal oxides, and transition metal oxides, which can be used to synthesize a variety of functional porous materials [5,6,7].

Porous materials are materials that contain rich pore structures. They exhibit good photoelectric, propagation, mechanical, adsorption, permeability, and chemical properties. They can be made from various raw materials, such as metals, carbides, borides, nitrides, and silicides. Porous materials based on Si are among the most widely used types of porous materials. Common Si-based porous materials include zeolites, aerogels, and ceramics; these materials are often produced by Si-bearing chemicals or minerals (kaolinite, bentonite, etc.) [8,9,10].

In recent years, the demand for new materials in the field of energy and environmental protection has increased due to national industrial upgrading and ecological protection needs. Si-based porous materials have a large specific surface area, adjustable structure, strong photoelectric performance, and excellent adsorption and catalytic properties [11,12,13,14]. They are essential in the field of energy and environmental protection. As environmental pollution is increasingly severe, many adsorbents are in demand. Si-based porous materials such as functional ceramics, aerogels, zeolites, and geological polymers are widely used to prevent and treat sewage and waste gas pollution due to their strong adsorption capacity [15,16]. As resources such as oil and natural gas are continuously depleted in the energy field, the necessity and urgency of using existing resources such as waste plastics and biomass for energy regeneration are constantly increasing. This indicates the huge application prospects of various Si-based catalysts [17]. In addition, porous silicon anode materials have many advantages in various new energy Li-ion battery electrode materials and have the potential to be used as raw materials for the next generation of Li-ion batteries [18].

However, limited raw material sources restrict Si-based porous materials’ production and application. Fortunately, recent studies have found ways to prepare functional Si-based porous materials from Si-rich coal-based solid waste such as coal gangue and fly ash [19,20]. Techniques such as foaming, autoclaving, high-temperature sintering, and hydrothermal synthesis are applied to convert coal-based solid waste into porous materials. The resulting products, including various solid-waste-based zeolites, ceramics, geopolymer, etc., have abundant pore structures, stable properties, and are made from inexpensive and readily available raw materials, which can be applied in practical production [21,22,23,24].

This article reviews the advanced processes for converting coal gangue and fly ash into various energy and environmental protection porous materials. It summarizes the strengths and weaknesses of these materials in wastewater treatment, waste gas treatment, energy catalysis regeneration, and battery manufacturing. The application prospects of coal-gangue-based and fly-ash-based porous materials are also discussed at the end of this article. The conclusions will reference the resource utilization of typical coal-based solid waste and contribute positively to achieving the country’s ‘carbon peaking and carbon neutrality goals’.

## 2. Physicochemical Characteristics and the Recycling of Coal Gangue and Fly Ash

### 2.1. Physicochemical Characteristics of Coal Gangue

Coal gangue is a waste material from coal mining and washing in the coal industry. Its main sources include shale, dirt band, and washery rejects. It is a black-gray rock with a lower carbon content and greater hardness than coal. The chemical composition of coal gangue mainly includes SiO_2_, Al_2_O_3_, Fe_2_O_3_, CaO, etc., as well as some organic components (mainly C) and trace rare metal elements. Table 1 shows the chemical composition of typical coal gangue (LOI is ‘loss on ignition’) [25]. The main mineral composition of coal gangue varies in different parts of the world. Chinese coal gangue mainly comprises kaolinite, quartz, illite, and a small amount of calcite and iron ore [25,26]. 

### 2.2. Physicochemical Characteristics of Fly Ash

Fly ash is a fine ash captured from the flue gas produced by coal combustion. It is the primary solid waste from coal-fired power plants. Depending on the method of boiler combustion, fly ash can be classified as either circulating fluidized bed (CFB) fly ash or pulverized coal (PC) fly ash [27].

A CFB boiler typically uses low-quality coal as fuel and needs to maintain a furnace temperature of 850–950 °C. The resulting fly ash primarily consists of irregularly structured slag-like particles. Due to the low furnace temperature, the interior still contains highly organic components such as sulfur and carbon. In terms of its phase composition, CFB fly ash mainly contains quartz, hematite, magnetite, and small amounts of calcite, periclase, and anorthite [28,29].

PC fly ash is formed by combustion and cooling of high heating value coal powder (particle size less than 100 μm) in a pulverized coal furnace at temperatures above 1400 °C. It is the main type of fly ash in China currently. Because of the high-temperature combustion and rapid cooling process, PC fly ash usually appears as spherical particles of various sizes. In terms of the chemical composition, most of the organic components in coal ash are completely decomposed by combustion at high temperatures. However, there are still traces of residual polycyclic aromatic hydrocarbons (PAHs) remaining after combustion, which have toxic, mutagenic, and carcinogenic properties [30]. Table 2 shows the chemical composition of typical fly ash in China [31]. In terms of phase composition, PC fly ash is mainly composed of spherical amorphous bodies (accounting for 50% to 80% of the total amount), including high-iron amorphous bodies, low-iron amorphous bodies, amorphous SiO_2_, and amorphous Al_2_O_3_. In addition to amorphous bodies, the mineral crystal composition is similar to CFB fly ash but includes some mullite phases formed at high temperatures [32].

### 2.3. General Processing Methods of Coal Gangue and Fly Ash

**Figure 2:**
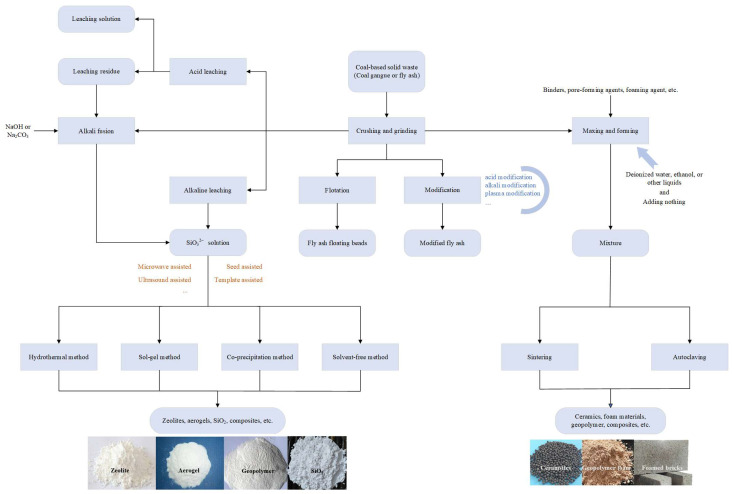
General processing methods of typical coal-based solid wastes (coal gangue and fly ash).

There are three main methods for the recycling and utilizing of coal gangue and fly ash by preparing Si-based porous materials [33]: (1) extracting valuable elements from coal gangue and fly ash through various physicochemical methods to achieve the reproduction of porous materials; (2) mixing coal gangue and fly ash with other active substances to produce porous materials; (3) directly modifying the solid waste based on its inherent characteristics to produce porous materials. The specific processes are shown in Figure 2.

#### 2.3.1. Extraction of Si and Reproduction

To effectively extract the abundant Si contained in various crystal phases of coal gangue and fly ash, it needs to be activated through multiple steps such as crushing, grinding, alkali fusion, and other processes. The silicon source liberated from coal gangue and fly ash can be synthesized into zeolites, aerogels, and other Si-based porous materials through processes such as the hydrothermal method, the sol–gel method, the co-precipitation method, microwave-assisted thermal crystallization, etc. [34]. For example, Zhu et al. [35] designed a three-step scheme of calcination-alkali fusion-acid dissolution to achieve efficient separation of Si from coal gangue and synthesized a low-density nanostructured aerogel. Ozdemir et al. [36] used the method of alkali fusion to extract the silicon source from fly ash and synthesized X-type zeolite through ultrasound-assisted hydrothermal synthesis.

Currently, the most effective process for synthesizing Si-based porous materials is to treat coal-based solid waste with acid leaching to remove impurities, followed by alkali melting and hydrothermal synthesis. This method can produce high-quality products with good performance, and it is one of the most commonly used methods in the field of solid waste resource utilization [23,34,37].

#### 2.3.2. Mixing with Active Substances

Coal gangue and fly ash powder can be mixed with binders, pore-forming agents, foaming agents, and other active substances to form various functional ceramics, foam materials, composites, and other porous materials through techniques such as high-temperature sintering, autoclaving, and foaming. For instance, Wang et al. [38] prepared porous cordierite ceramic through slurry preparation, sponge selection, sponge immersion, drying, and sintering, using the raw materials mixed with 66.2 wt% coal gangue, 22.7 wt% basic magnesium carbonate, and 11.1 wt% bauxite. Using coal gangue powder and Al(OH)_3_ as the main raw materials, with MoO_3_ as an additive, a high-porosity mullite ceramic membrane could be prepared by sintering at 1400 °C [39]. Mixing fly ash with active substances into a slurry and then sintering the slurry in the furnace or autoclaving them in the reactor is also a method to produce porous materials [40,41].

#### 2.3.3. Direct Modification

Commonly, there are a large number of bead-like particles in the fly ash. Due to the gasification of volatile components during the combustion of coal powder, the formed fly ash contains pore structures [42]. Therefore, fly ash itself is a kind of usable porous material. By modification, fly ash can form a pore structure and rougher surface, greatly increasing its specific surface area and effectively enhancing its performance in adsorption, catalysis, and other aspects. The common modification methods include acid modification, alkali modification, and plasma modification [43,44,45].

Among the various bead-like particles in fly ash, a thin-walled hollow bead can be obtained through fly ash flotation. This is a hollow spherical amorphous body with uniform particle size, corrosion resistance, high temperature resistance, and a large specific surface area. The fly ash floating beads can be used as excellent carriers to process into various porous materials [46].

## 3. Application of Solid-Waste-Based Porous Materials in the Field of Energy and Environmental Protection

### 3.1. Application of Solid-Waste-Based Porous Materials in the Field of Wastewater Treatment

The discharge of industrial wastewater has long been a concern for the state. Industries such as electroplating, printing and dyeing, smelting, and pharmaceuticals produce large amounts of wastewater annually. The various types of waste water can severely harm the ecology. To solve the increasingly severe water pollution problem, functional porous materials such as solid-waste-based zeolites, solid-waste-based ceramic particles, solid-waste-based Fenton-like catalysts, solid-waste-based composite photocatalysts, etc., have been developed and applied to treat heavy metal ions, ionic dyes, and organic pollutants in wastewater [47,48,49,50]. These materials have physical adsorption capabilities and can efficiently treat specific pollutants through their active components.

#### 3.1.1. Adsorbing Heavy Metal Ions and NH_4_^+^ through Ion Exchange of Zeolites

Ion exchange is the process of exchanging between ions on an ion exchanger and ions with the same charge in a solution. Zeolite, a traditional porous ion exchanger, has been used for adsorbing various cationic pollutants in wastewater for a long time. Zeolite material consists of SiO_4_ and AlO_4_ frameworks. Since Al is trivalent, an excess negative charge is needed around it to form an AlO_4_ structure, making the entire material negatively charged. The compensating cations outside the silicon–aluminum framework, which are used to maintain electrical neutrality, can exchange with heavy metal ions in wastewater to remove them [51]. Low-cost zeolite materials made from fly ash and coal gangue can effectively adsorb heavy metal ions in wastewater.

**Figure 3:**
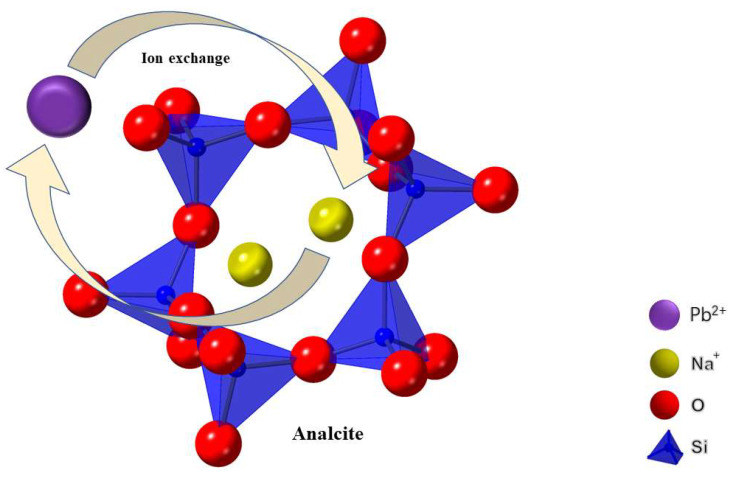
Adsorption mechanism of Pb^2+^ by the analcite-activated carbon composite [47].

Li et al. [47] created an analcite-activated carbon composite from coal gangue and investigated its adsorption mechanism of Pb^2+^ (Figure 3). The research showed that the specific surface area of analcite-activated carbon composite was 20.8202 m^2^/g. It could rely on ion exchange to adsorb Pb^2+^ with a maximum adsorption capacity of 125.57 mg/g. Ankrah et al. [52] used fly ash to synthesize mesoporous NaP1 zeolite and achieved the adsorption of multiple metal ions such as Zn^2+^, Cu^2+^, and Pb^2+^. Besides metal ions, NH^4+^ in wastewater can also be adsorbed similarly. By mixing fly ash, red mud, and coffee grounds with water, a slurry was shaped and sintered into ceramsite. Then, making the NaP zeolite crystallized on the surface of the ceramsite by hydrothermal treatment with NaOH and NaAlO_2_ at 373 K for 24 h, a hierarchically porous NaP zeolite composite was obtained with an NH^4+^ adsorption capacity of 13.1 mg/g at 318 K [53]. Chen et al. [54] used a hydrothermal method to convert fly ash into highly porous converted CFA, with X-zeolite as the main active phase. Through adsorption experiments, it was found that this material’s maximum adsorption capacity for NH^4+^ was 138.89 mg/g.

Treating heavy metal ions and NH^4+^ based on the ion exchange of solid-waste-based zeolite has advantages such as low cost, large adsorption capacity, simple operation, and good regeneration performance. However, there are also some limitations. Ion exchange must be carried out at an appropriate pH value. A lower pH value will cause H^+^ in the wastewater to participate in ion exchange, and a higher pH value will cause metal ions (such as Pb^2+^, Cu^2+^, etc.) or NH^4+^ to react with OH^−^. Additionally, the difficulty of adsorption depends on the ion exchange affinity of different ions in the wastewater. Generally, the smaller the ion radius and the more positive charge carried by the ion, the greater the ion exchange affinity and the easier the adsorption.

#### 3.1.2. Adsorption of Metal Ions and Ionic Dyes Based on the Interaction of Hydroxyl Groups on the Material Surface

The surface of Si-based porous materials is rich in hydroxyl groups (≡Si-OH). In an acidic medium, these hydroxyl groups decompose into ≡Si^+^, making the material surface easier to adsorb anions. In an alkaline medium, they decompose into ≡Si-O^−^, making the material surface easier to adsorb cations. Due to this electrostatic attraction, various Si-based porous materials produced from coal gangue and fly ash can effectively adsorb metal ions and ionic dyes.

**Figure 4:**
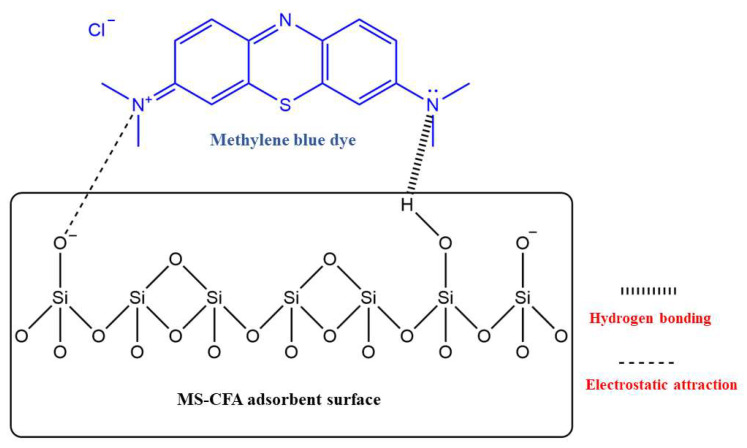
Adsorption mechanism of methylene blue dye by the MS-CFA [56].

Sahoo et al. [55] modified fly ash (MFA) with simple alkali soaking and used it to adsorb heavy metal ions in acidic mine wastewater. The surface area of MFA increased nearly two times through modification. The results showed that the rich functional oxygen-containing groups (≡Si-OH and ≡Al-OH) on the surface of the MFA played a positive role in metal ion adsorption, and the removal rate of heavy metal ions in the treated wastewater can reach over 90%. Compared to metal ions, ionic dyes have larger volumes and complex compositions of various functional groups, which can easily interact with the hydroxyl groups on the material surface. Sareen et al. [56] created a coral-reef-shaped mesoporous silica material (MS-CFA) with a specific surface area of 350 m^2^/g from calcined fly ash by acid leaching, alkali melting, precipitation, and calcination and studied its adsorption mechanism of methylene blue (a cationic dye) (Figure 4). Through adsorption experiments, it was observed that the adsorption rate increased with the pH due to electrostatic attraction and decreased due to the weakening of hydrogen bonds because of the combination of excessive OH^−^ and amino groups on methylene blue when the pH value reached 9, further demonstrating the mechanism. The results showed that MS-CFA could adsorb up to 20.49 mg/g of methylene blue under optimal conditions. The same conclusion was obtained by Yan et al. in their analysis of the adsorption mechanism of methylene blue and magenta dyes on coal-gangue-based porous ceramic microspheres; that is, the adsorption behavior of ionic dyes based on the surface hydroxyl interaction of Si-based porous materials is caused by electrostatic attraction, hydrogen bonding, and other forces. The maximum adsorption capacity of this ceramic microsphere for methylene blue reached 30.01 mg/g, while for magenta, it reached 24.16 mg/g [48].

**Figure 5:**
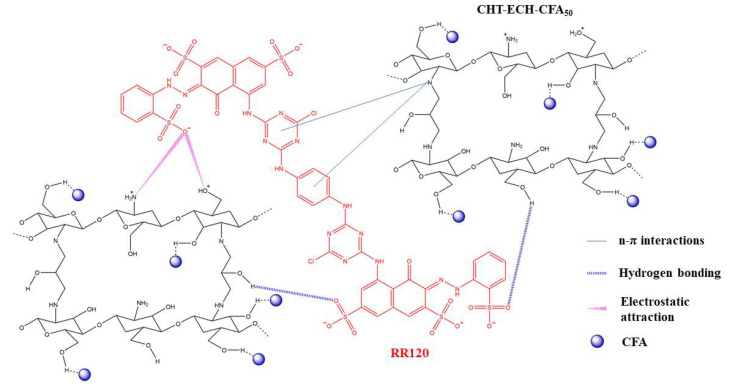
Interaction between CHT-ECH/CFA50 surface and RR120 dye: electrostatic attraction, hydrogen bonding interaction, and n-π interaction [57].

Loading substances rich in hydroxyl groups onto porous materials can further enhance their dye adsorption performance. Mohammed et al. [57] dissolved chitosan (CHT) in acetic acid and adjusted the pH value to form CHT microspheres. The CHT microsphere solution and coal fly ash (CFA) were added to the epichlorohydrin (ECH) solution to produce a biocomposite adsorbent of covalently crosslinked chitosan–epichlorohydrin/coal fly ash (CHT-ECH/CFA_50_). The specific surface area of CHT-ECH increased significantly by loading with CFA, and CHT contained a large number of hydroxyl groups, amino groups, and N atoms with lone pair electrons; hence, it had a variety of strong adsorption effects on the Reactive Red 120 dye (RR120) (Figure 5). The maximum adsorption capacity of CHT-ECH/CFA_50_ for RR120 dye can reach 237.7 mg/g.

Under conditions of suitable pH, Si-based porous materials made from Si-rich solid wastes such as coal gangue or fly ash can effectively adsorb metal ions and ionic dyes through various adsorption effects generated by surface hydroxyl groups. These materials can be regenerated through chemical elution and other methods. Currently, Si-based materials represented by zeolites can basically achieve the adsorption treatment of heavy metal ion pollutants through the electrostatic attraction and ion exchange effects of surface hydroxyl groups. However, it is difficult to achieve a large amount of adsorption of large molecular ionic dyes just by the limited functional hydroxyl groups on the surface of Si-based porous materials and their small and narrow pore structure. Preparing modified materials by loading substances that are rich in functional active groups, such as chitosan, onto porous materials has become the main development direction of ionic dye adsorbents.

#### 3.1.3. Precipitation and Adsorption of the Pollutants Based on the Alkali Supply Capacity of Materials

Mixing fly ash and coal gangue with other active substances through high-temperature sintering or autoclaving can be made into porous materials that can continuously and stably release OH^−^ into water, allowing pollutants such as Mn and P, which easily form insoluble substances in an alkaline environment, to be precipitated and adsorbed onto the surface of the material.

Ou et al. [58] mixed fly ash and lime cement to prepare CFA-based ceramsite with an apparent porosity of 49.49%. By studying and analyzing the XRD patterns of the ceramsite before and after the adsorption of Mn^2+^, the alkali supply mechanism of this type of material was proposed. After the ceramsite adsorbed Mn^2+^, diffuse peaks appeared in its XRD patterns in the range of 2θ = 10~15°, indicating the formation of a small amount of amorphous phase. The reason is that the anorthite and gehlenite with lattice imperfections underwent a hydration reaction, as shown in Reactions (1) and (2):

CaAl_2_Si_2_O_8_ + 3H_2_O = Ca^2+^ + 2OH^−^ + Al_2_[Si_2_O_5_][OH]_4_ (1)

2Ca_2_Al_2_SiO_7_ + 9H_2_O = 4Ca^2+^ + 6OH^−^ + 2Al[OH]_4_^−^ + Al_2_[Si_2_O_5_][OH]_4_ (2)

The progress of the reaction promotes the formation of an alkaline environment, causing Mn^2+^ to react with OH^−^ to form Mn(OH)_2_ precipitate, which adheres to the surface of the ceramsite. The ability of CFA-based ceramsite to release OH^−^ is stable and persistent.

Phosphorus removal also has a good effect based on a similar method, and the Ca^2+^ released by the hydration reaction could also be consumed simultaneously during the formation of calcium phosphate salt precipitation. Using cement, clay, calcium oxide, and fly ash as the main raw materials and aluminum powder as the pore-forming agent, after mixing and forming, the TBX porous ceramsite could be obtained by autoclaving in a high-pressure reaction kettle. The experimental results show that under the influence of TBX ceramsite’s high alkali supply and calcium release ability, the removal rate of phosphorus can reach more than 98% [41].

Using solid-waste-based porous materials with alkali supply capacity for precipitation adsorption treatment of wastewater has advantages such as low cost, simple operation, and convenient treatment. Furthermore, a large amount of pollutants are concentrated and adsorbed on the surface of the porous material, which is conducive to their recycling and utilization. However, during water treatment, long-term deposition can cause the material surface to be covered by pollutant precipitation, greatly affecting the material’s adsorption and alkali supply performance and limiting the application of such materials.

#### 3.1.4. Loading Photocatalytic Materials for the Degradation of Organic Pollutants

Common photocatalytic materials such as ZnO, Fe_2_O_3_, and TiO_2_ can produce a large number of electron holes (h^+^) and electrons (e^−^) in a short amount of time to assist in the degradation of organic matter under the irradiation of a specific wavelength light. Due to the low quantum activity of the photocatalytic process, these materials are often made into nanoparticles to improve their photocatalytic activity. These particles are also prone to agglomeration during the photocatalytic degradation of organic matter, causing deactivation [59]. To improve the photocatalytic activity and service life, it is a good method to load these nanoparticles onto porous materials to make composite photocatalysts. Various Si-based porous materials made from fly ash and coal gangue are good carriers for photocatalytic materials.

Wang et al. [49] obtained a modified SiO_2_-Al_2_O_3_ aerogel from aluminosilica-sol (produced by alkali melting and acid leaching of fly ash) through gel, aging, solvent exchange, surface modification, washing, and drying. Ti-oxide nanoparticles were loaded onto the aerogel using the sol–gel method to create TiO_2_/SiO_2_-Al_2_O_3_ aerogel composites used for the photocatalytic degradation of 4,6-dinitro-2-sec-butylphenol (DNBP). The test results showed that the loading of TiO_2_ nanoparticles increased the reaction contact area of TiO_2_, improved photocatalytic efficiency, and prolonged the service life of the photocatalyst. Stirring modified coal gangue with rich micropores in ZnSO_4_ solution for 1 h, after adjusting the pH value of the solution to 9 and boiling refluxing for 3 h, a ZnO/coal gangue composite was prepared for the photocatalytic degradation of methyl orange and methylene blue organic dyes. The degradation of organic dyes was basically achieved within 120 min, and the degradation rate of this catalyst remained above 65% after repeated use for five times [60]. Bai et al. [61] mixed fly ash floating beads with butyl titanate and stirred for a while. After drying and calcining, a TiO_2_/fly ash floating bead composite photocatalyst was obtained. Under the conditions of UV light irradiation for 2 h, pH 10.0, and TiO_2_ loading amount of 28.57%, the degradation rate of Rhodamine B organic dye was over 80% and had good recycling regeneration.

Several studies have shown that fly ash and coal-gangue-based composite photocatalysts have advantages such as easy availability of raw materials, green and sustainable driving energy, long service life, good regeneration performance, and a wide application range. However, it is difficult to achieve complete degradation of pollutants in wastewater with high COD (chemical oxygen demand) values only by the photocatalytic process. In addition, many photocatalytic materials only exhibit high catalytic degradation performance under specific wavelength light irradiation, further increasing the cost of use. The pH value can also affect the energy level structure of photocatalytic materials and impact the photocatalytic process.

#### 3.1.5. Preparing Heterogeneous Fenton-Like Catalysts for the Degradation of Organic Pollutants

The Fenton reaction is an oxidation process of organic compounds to inorganic states in a mixed solution of Fe^2+^ and hydrogen peroxide. The specific process can be represented by the following reaction [62]:

Fe^2+^ + H_2_O_2_ → Fe^3+^ + OH^−^ + OH· (3)

H_2_O_2_ + 2Fe^3+^ → 2Fe^2+^ + HO_2_· + H^+^ (4)

O_2_ + Fe^2+^→ Fe^3+^ + O_2_^−^ (5)

The strong oxidation of Fenton reagents comes from the hydroxyl radicals (OH·) and peroxide radicals (HO_2_·) generated in the reaction. Based on the principle of this process, using iron-containing minerals, Fe^3+^, or transition metals such as Cu, Co, Cd, Ni, etc., to generate strong oxide from H_2_O_2_ is called a Fenton-like reaction [63]. The conventional Fenton reaction is a homogeneous reaction with strict requirements for conditions such as pH value and H_2_O_2_ addition amount, and it is difficult to recover the catalyst after the reaction [64]. Connecting active substances such as Fe to the porous materials to prepare heterogeneous Fenton-like catalysts can significantly reduce the loss of active ingredients and make the reaction conditions milder [65]. Fly ash contains rich iron oxides and has a porous structure that is conducive to the adsorption of organic pollutants, making it an excellent resource for preparing heterogeneous Fenton-like catalysts.

**Figure 6:**
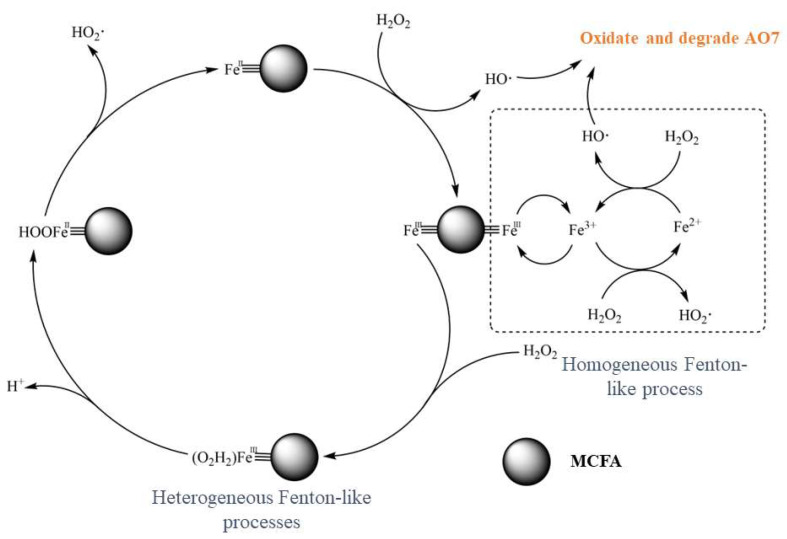
Catalytic mechanism of MCFA in Fenton-like processes [50].

Wang et al. [50] studied the catalytic mechanism of H_2_SO_4_-modified coal fly ash Fenton-like catalysts (MCFA) in degrading Acid Orange 7 (AO7) (Figure 6). It was discovered that in addition to the homogeneous catalysis process, a small amount of Fe^3+^ dissolved in water also carried out a homogeneous catalysis process simultaneously. Experimental results demonstrated that under optimal conditions, the removal rate of AO7 exceeded 95%, and after being reused six times, its removal rate remained around 90%. Coal fly ash was crushed, sieved, and dried by Chen and Du to produce a Fenton-like catalyst for degrading n-butyl xanthate. Under the conditions of reaction temperature of 30 °C, reaction time of 120 min, pH 3.0, H_2_O_2_ concentration of 1.176 mmol/L, catalyst concentration of 1.0 g/L, and Fe (III) content of 4.14%, the removal rate of n-butyl xanthate exceeded 96.90% [66].

**Figure 7:**
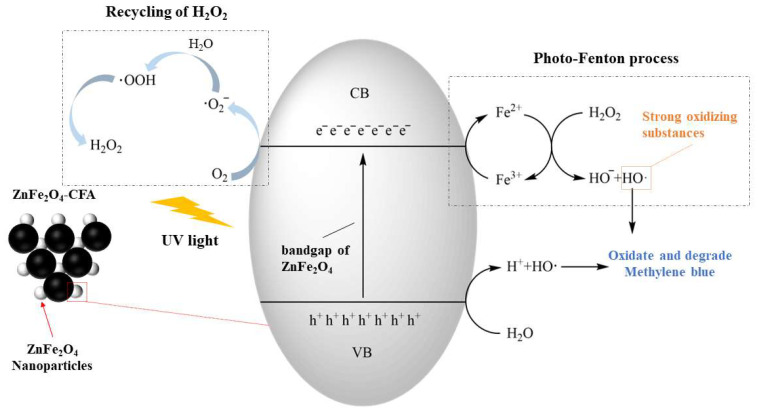
Synergistic mechanism of photocatalysis and Fenton-like process by CFA-ZnFe_2_O_4_ composite [67].

Photo-Fenton technology is a technique that employs photocatalysis to assist the Fenton reaction. Compared to traditional Fenton processes, photo-Fenton technology possesses the advantages of both, and it is more effective in treating organic pollutants. Nadeem et al. [67] added hydrothermally treated coal fly ash (CFA) into a ZnFe_2_O_4_ precursor solution and synthesized ZnFe_2_O_4_-CFA composite Fenton-like catalysts through hydrothermal synthesis. SEM-EDS analysis shows that the surface of composites is very rough and the zinc ferrite is uniformly loaded, making it a photo-Fenton catalyst with good adsorption performance. Figure 7 illustrates the mechanism flowchart of ZnFe_2_O_4_-CFA composite degrading methylene blue dye under UV light. ZnFe_2_O_4_ generates h^+^ and e^−^ under UV light, where h^+^ reacts with H_2_O to form H^+^ and OH· while e^−^ forms Fe^2+^ with Fe^3+^. The resulting Fe^2+^ undergoes a Fenton reaction with H_2_O_2_ to form OH^−^and OH·. The formed OH· will oxidize and degrade methylene blue due to its strong oxidizing property. In addition, the O_2_ produced during the reaction generates superoxide anions (·O_2_^−^) with e^−^, which further oxidize into H_2_O_2_, achieving the recycling of H_2_O_2_ (Equations (6)–(10)):

HO_2_· + OH· → H_2_O + O_2_ (6)

H_2_O_2_ + 2h^+^ → O_2_ + 2H^+^ (7)

O_2_ + e^−^ → ·O_2_^−^ (8)

H_2_O + O_2_·^−^→ ·OOH + OH^−^ (9)

2·OOH → O_2_ + H_2_O_2_ (10)

The results showed that the ZnFe_2_O_4_-CFA composite (mCFA:mZnFe_2_O_4_ = 1:1) had a degradation rate of up to 97% for methylene blue. After being used five times, the degradation rate remained above 85%. ZnFe_2_O_4_-CFA composite is a superior photo-Fenton catalyst with the characteristics of Fe not easily leaching out and renewable reactants.

As a green and efficient water treatment method, Fenton oxidation technology can achieve deep oxidation of wastewater by generating a large amount of OH· and greatly reducing the COD value in the water. The development and appliance of various solid-waste-based heterogeneous Fenton-like catalysts can effectively reduce costs and greatly improve the disadvantages of traditional homogeneous Fenton catalysts, such as limited reaction conditions and poor regeneration. Advanced methods such as light- and electricity-assisted Fenton reactions have further enhanced the degradation efficiency of pollutants. It should be considered that pH value and H_2_O_2_ concentration are still the main factors affecting the catalytic performance of Fenton-like catalysts due to the limitations of their catalytic mechanism.

The development and appliance of coal-gangue-based and fly-ash-based water treatment materials can effectively reduce raw material costs while properly disposing of a large amount of accumulated coal-based solid waste. Different types of solid-waste-based materials can efficiently adsorb specific pollutants in wastewater based on their unique physicochemical properties and mechanisms of action, and they have unique advantages, such as the green sustainability of composite solid-waste-based photocatalysts and the high-efficiency versatility of solid-waste-based Fenton-like catalysts. Besides this, limited by the action mechanism of different solid-waste-based water treatment materials, external conditions such as pH value, pollutant type, illumination, etc., greatly impact the water treatment performance of the materials. In practical applications, it is necessary to choose suitable materials for treatment according to the specific situation. In addition, solid-waste-based water treatment materials are in contact with water for a long time during the treatment of wastewater. Harmful components such as heavy metal ions in solid waste will slowly escape, causing secondary pollution (especially for structurally unstable materials).

### 3.2. The Application of Solid-Waste-Based Porous Materials in the Field of Waste Gas Treatment

Common exhaust gases include CO_2_, SO_2_, NO_x_, and volatile organic compounds (VOCs). These gases can directly harm animals and plants, indirectly damage the ecological environment by polluting the atmosphere, or contribute to the greenhouse effect, causing global warming and rising sea levels. The adsorption method is a mature exhaust gas treatment technology with advantages such as low energy consumption, high efficiency, and a simple process. Various coal-gangue-based and fly-ash-based gas adsorbents have been developed for the adsorption treatment of exhaust gases [68,69,70]. The diameter of gas molecules is generally on the order of 0.1 nm. Strong physical adsorption can be achieved by controlling the formation of a rich and evenly distributed mesoporous structure in solid-waste-based adsorbents [71]. Additionally, by active components contained in solid waste or obtained through loading modification, solid-waste-based materials can further produce various forces on exhaust gases, greatly enhancing the performance of these materials.

#### 3.2.1. The Adsorption of CO_2_ Based on Hydroxyl Hydrogen Bonding

**Figure 8:**
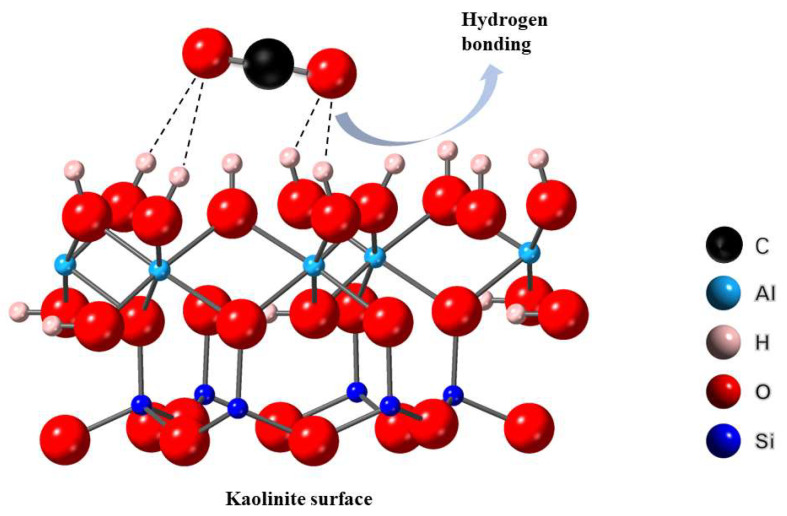
Adsorption model of CO_2_ on kaolinite surface [72].

Hydroxyl groups on the surface of solid materials can adsorb CO_2_. Some researchers have investigated the adsorption mechanism of CO_2_ by hydroxyl groups on solid surfaces. The O atom of CO_2_ can form hydrogen bonds with hydroxyl groups (as shown in Figure 8, taking kaolinite as an example) [72]. The surface of Si-based gas adsorbents made from coal gangue or fly ash is rich in hydroxyl groups, making these adsorbents ideal materials for CO_2_ adsorption treatment.

Yan et al. [68] prepared an alkaline silicate material with a mesoporous structure from fly ash and found that the material’s surface was rich in hydroxyl groups through FT-IR analysis. They established an isotherm modeling of CO_2_ using adsorption–desorption experiments. They found that it conformed to the Langmuir isotherm model, indicating strong adsorption forces on its surface, and the partial desorption of CO_2_ after heating confirmed the existence of hydrogen bonding. Wu et al. [73] hydrothermally synthesized Mg-doped CuSiO_3_ material by adding a Mg^2+^ solution to a SO_3_^2−^ solution made from coal gangue. Although the doping of Mg leads to a decrease in the pore size and specific surface area of the mesoporous CuSiO_3_ material, it could effectively increase the number of hydroxyl groups on the surface of the CuSiO_3_ material. The experimental results showed that within a certain range, the higher the doping amount of Mg, the higher the CO_2_ adsorption capacity of Mg-doped copper silicate material, and that the maximum adsorption capacity could reach up to 8.38 mg/g.

Due to the instability of H-bonds at high temperatures, the adsorbed CO_2_ can be desorbed simply by heating. Therefore, porous materials with adsorption capacity based on H-bonding have good regenerability. However, the adsorption efficiency of such materials is extremely low in high-temperature environments, and since H_2_O molecules can also form H-bonds with -OH groups, temperature and humidity are both important factors that affect the CO_2_ adsorption process.

#### 3.2.2. Introducing Amino Groups by Chemical Modification for CO_2_ Adsorption

Amine groups can interact with CO_2_ molecules through the production of ion intermediates. The specific process is as follows [74]:

CO_2_ + 2RNH_2_ → RNHCOO^−^ + RNH^3+^ (11)

Introducing amine groups into solid-waste-based gas adsorbents through chemical modification methods can effectively improve their CO_2_ adsorption performance.

Chandrasekar et al. [69] extracted silicon and aluminum sources from fly ash through alkali fusion. Pluronic P123 (EO_20_PO_70_EO_20_) was added as a template agent to synthesize the SBA-15 zeolite with an average pore size of 7.2 nm. Polyethyleneimine (PEI) was introduced into the zeolite through wet impregnation to produce a PEI-loaded SBA-15 zeolite with rich amino groups on its surface. The experiments showed that this material can adsorb up to 110 mg/g of CO_2_ at 75 °C. Using SiO_3_^2−^ leachate produced from coal gangue as a silicon source and CTAB as a template agent, a M-SiO_2_ mesoporous silica material with MCM-41 structure was synthesized. An EDA-M-SiO_2_ porous material was obtained by impregnating in ethylenediamine (EDA) solution and drying, and the material exhibited strong CO_2_ adsorption performance, with an adsorption capacity of 83.5 mg/g under optimal conditions [75].

The introduction of amino groups greatly improves the CO_2_ adsorption capacity of solid-waste-based gas adsorbents. Since CO_2_ interacts with the adsorbent through chemical reactions, these materials have higher stability. However, excessive loading of amine substances can block pore channels and greatly reduce the adsorption performance of the porous material. In addition, H_2_O molecules can easily form hydrogen bonds with amino groups, so humidity can affect adsorption.

#### 3.2.3. The Absorption of CO_2_ and SO_2_ Based on Alkaline Substances of Solid Waste

Alkaline substances such as CaSiO_3_, K_2_SiO_3_, CaO, and MgO can effectively absorb acidic gases such as CO_2_ and SO_2_ through chemical reactions. Coal-based solid wastes such as fly ash and coal gangue contain some of these substances or the raw materials needed to produce them [25,31].

Sanna et al. [76] mixed fly ash (FA) with a certain amount of K_2_CO_3_ (K) and calcined it to obtain a K-FA porous material with K_2_SiO_3_ as the main active substance for CO_2_ adsorption; 10 wt% Li_2_CO_3_ was added to form the K-Li eutectic phase, improving the internal diffusion performance of the K-FA material and accelerating the CO_2_ adsorption–desorption process. This material mainly absorbs CO_2_ through the following reaction:

K_2_SiO_3_ + CO_2_ → K_2_CO_3_ + SiO_2_ (12)

It was measured that at 700 °C, the CO_2_ adsorption capacity of the K-FA material (nFA: nK = 1:1, 10 wt% Li_2_CO_3_) was 104.72 mg/g.

By adding a certain amount of Ca(OH)_2_ solution to the alkaline-leached silicon solution from fly ash, filtering, and drying the mixture after reacting, an active calcium silicate material (ACS) would be prepared for SO_2_ absorption. BET analysis showed that this material contained abundant mesoporous structures. In an adsorption experiment at 50 °C, it was found that the adsorption capacity of this material would be greatly increased when O_2_ and water vapor were present in the flue gas. The reason for this is that the ACS will react with SO_2_ to promote chemical adsorption when O_2_ and H_2_O are present. The process is as follows (12)–(17):

Where (g) and (ad) denote the gaseous and adsorbed states.

SO_2_(g) → SO_2_(ad) (13)

SO_2_(g) + H_2_O → H_2_SO_3_ (14)

SO_2_(ad) + H_2_O → H_2_SO_3_ (15)

H_2_SO_3_ + CaSiO_3_ → CaSO_3_ (16)

CaSO_3_ + 1/2H_2_O + 1/2O_2_ → CaSO_4_·1/2H_2_O (17)

CaSO_3_ + 1/2O_2_ → CaSO_4_ (18)

The maximum adsorption capacity of ACS for SO_2_ can reach 38.2 mg/g. After adsorption, the ACS can be reused by regenerating with water vapor at 300 °C for 3 h. After 20 regeneration cycles, its adsorption performance only decreased by 28.8% [77].

In summary, the solid-waste-based porous materials based on alkaline substances to absorb acid waste gas have good adsorption performance and can function in high-temperature and high-humidity environments. Nevertheless, this type of material requires long-term high-temperature regeneration when recycling, consuming more energy.

#### 3.2.4. Denitration Based on NH_3_-SCR Technology

NH_3_ selective catalytic reduction (NH_3_-SCR) technology is a process that selectively reduces NO_x_ to N_2_ within a certain temperature range assisted by catalyst, using NH_3_ as a reducing agent. It is one of the main research directions of denitrification technology at present. The main reaction is as follows:

4NO + 4NH_3_ + O_2_ → 4N_2_ + 6H_2_O (19)

Common NH_3_-SCR catalysts include transition metal catalysts such as Cu-based and Fe-based catalysts [78]. Some studies have found that metal-loaded zeolite catalysts have higher reaction activity and a broader active temperature range for the NH_3_-SCR process than metal/metal oxide catalysts [79]. These catalysts also exhibit good hydrothermal stability [80]. Therefore, preparing zeolite carriers from coal-based solid waste and loading metals onto them to synthesize denitration catalysts is an effective method for solid waste’s reuse.

**Figure 9:**
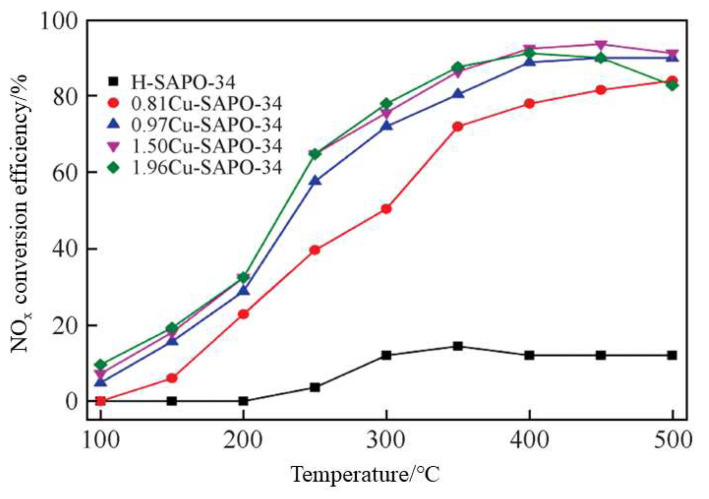
NH_3_-SCR activity of Cu-SAPO-34 catalyst with different Cu loading amounts [70].

Ma et al. [70] extracted silicon and aluminum sources from fly ash and produced SAPO-34 zeolite with a specific surface area of 579 m^2^/g and an average pore size of 0.56 nm by template method, and then loaded Cu onto SAPO-34 using the ion exchange method to obtain the Cu-SAPO-34 catalyst. Figure 9 shows the denitration activity of Cu-SAPO-34 with different Cu loading amounts between 100 and 500 °C. The results indicate that the synthesized Cu-SAPO-34 exhibit high NO_x_ conversion rates, good high-temperature activity, and anti-sintering properties. Similarly, the SSZ-13 zeolite was obtained through one-step hydrothermal synthesis using coal gangue as a raw material, and a certain amount of Cu was loaded to obtain the micropore Cu-SSZ-13 catalyst, which exhibits high denitration activity between 180 and 400 °C, with NO_x_ conversion rates exceeding 90%, and high hydrothermal stability [81].

Solid-waste-based NH_3_-SCR zeolite catalysts have numerous acidic sites on their surface that adsorb NH_3_ and promote the formation of reaction intermediates, making them possess excellent denitration performance. However, substances (such as H_2_O) that cause competitive absorption with NH_3_ can influence the catalytic performance of these catalysts. Additionally, SO_2_ can react with NH_3_ and O_2_ to form ammonium salts deposited on the catalyst surface or react with the metal loaded onto the catalyst to form sulfates, causing catalyst deactivation [82].

#### 3.2.5. Absorption of VOCs

Volatile organic compounds (VOCs) are diverse and complex in composition and can directly involve in atmospheric photochemical reactions with NO_x_ and other pollutants to produce ozone and secondary organic aerosols [83]. Due to significant differences in the physical and chemical properties of different VOCs, VOC adsorbents can only adsorb a few specific volatile organic compounds.

Yuan et al. [84] used high-alumina fly ash as raw material to produce a highly stable silicate material with a specific surface area of 98.47 m^2^/g and an average pore size of 31.42 nm. Dynamic adsorption experiments demonstrated that this material had a high toluene adsorption capacity and a low formaldehyde adsorption capacity. Toluene is adsorbed through van der Waals forces, while formaldehyde is adsorbed through hydrogen bonding with hydroxyl groups on the surface of the silicate material.

Using fly ash and coal gangue as raw materials to produce solid-waste-based gas adsorbents has significant economic and environmental benefits. Various solid-waste-based adsorbents have recently been developed to effectively adsorb common waste gases such as CO_2_, SO_2_, and NO_x_ [73,77,81]. Some solid-waste-based materials can strongly interact with specific waste gases through their active substances and functional groups, such as the reaction between alkaline substances in solid waste materials and acidic gases and the hydrogen bonding interaction between hydroxyl groups on the surface of solid waste materials and CO_2_. Introducing high-activity sites onto solid-waste-based adsorbents through impregnation, ion exchange, and other processes can further increase their adsorption capacity and broaden their range of applications. For instance, they are introducing amino groups onto solid waste adsorbents to increase their CO_2_ adsorption capacity, introducing transition metals to obtain denitration capability. However, the adsorption performance of solid-waste-based gas adsorbents is greatly influenced by external factors such as temperature, humidity, gas type, etc., due to the limitations of their adsorption mechanisms, resulting in a significant reduction in the adsorption rate. Additionally, some materials that absorb waste gas through chemical reactions face challenges in regeneration.

### 3.3. The Application of Solid-Waste-Based Porous Materials in Energy Regeneration

Over the last decade, the consumption of various non-renewable energy sources has been constantly increasing. In addition to finding renewable energy sources as alternatives, existing resources such as waste plastics, waste oil, and biomass can also be regenerated through catalytic cracking, hydrocracking, steam reforming, and other methods [85,86,87]. To achieve high-quality and efficient regeneration of energy, the use of catalysts is necessary. Materials such as zeolite and silica–alumina gels made from coal-based solid waste are typical solid acid catalysts. Solid alkali components such as CaO and MgO and transition metals such as Fe in solid waste can serve as catalytic active centers. Solid waste with porous structures, such as fly ash, also has the potential to serve as catalytic carriers. Solid-waste-based catalysts have considerable application prospects in the field of energy recovery. Table 3 summarizes the research status of preparing energy regeneration catalyst materials that are prepared from coal gangue and fly ash solid waste in recent years.

Overall, coal-gangue- and fly-ash-based catalysts have wide applications in the field of energy regeneration. They can effectively reduce the activation energy required for the regeneration of various resources such as waste plastics, biomass, and gas and improve product yield and quality. The alkali metal oxide and transition metal oxide components in solid waste have high catalytic activity for reactions such as cracking, steam reforming, and dry reforming. The SiO_2_ and Al_2_O_3_ components can not only serve as catalyst carriers to enhance the stability of the catalyst and improve the reaction activity, but they can also synthesize zeolite materials through alkaline fusion and hydrothermal processes to further promote the cracking reaction (solid acid mechanism). Moreover, synthesizing zeolite products is more conducive to the structural adjustment and active metal loading of the catalyst, making solid-waste-based catalysts have higher regeneration efficiency and wider applications. However, the energy regeneration process is often accompanied by a high-temperature and high-carbon environment, which can easily cause coking and carbon deposition on the catalyst, leading to its deactivation. In addition, solid waste raw materials are complex in composition, and it is difficult to explore the specific mechanisms of each component in the catalytic process, making it difficult to control the regeneration process and achieve its large-scale application.

### 3.4. The Application of Solid-Waste-Based Porous Materials in Battery Manufacturing

As a new energy system, Li-ion batteries have advantages over traditional batteries, such as high energy density, good stability and cyclicality, low self-discharge rate, and voltage hysteresis [103]. Currently, graphite with good conductivity and a low cost is widely used as the anode material for Li-ion batteries. It can combine with Li to form LiC_6_ with a theoretical capacity of 372 mAh·g^−1^. Si-based anode materials are preferred for the next generation of high-capacity Li-ion battery anodes. At room temperature, every 4 Si atoms can react with 15 Li ions to form an alloy phase with a theoretical capacity of 3579 mAh·g^−1^, and Si-based materials have advantages such as environmental friendliness, abundant sources, and low cost [104,105]. However, the alloying reaction between Si and Li during repeated charging and discharging processes can cause severe volume expansion, resulting in severe battery loss [106]. Studies have shown that the nanostructure design can effectively alleviate the stress changes during the charging and discharging process of Li–Si alloys to maintain the maximum capacity of the battery [107]. At present, there have been several reports on the preparation of porous nano-silicon particles from silicon-rich coal-based solid waste as anode materials for Li-ion batteries.

**Figure 10:**
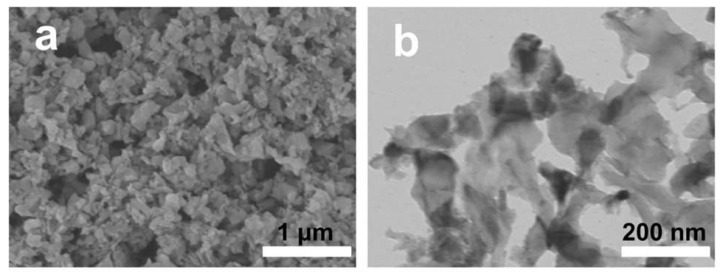
(**a**) SEM; (**b**) TEM image of Si nanoparticles [108].

Liu et al. [108] used Si-rich fly ash as a raw material to produce SiO_2_ through a process of alkali fusion, deionized water dissolution, acidification precipitation, washing and drying, and calcination. Finally, low-cost Si particles were obtained through a magnesiothermic reaction for use as anode materials. The resulting Si particles were nanoscale in size and interconnected to form a porous structure (Figure 10). The Li-ion battery made using these silicon nanoparticles as anode material provided a capacitance of approximately 3173.1 mAh·g^−1^ and remained stable after 500 cycles at 1 °C. A similar method was used by Xing et al. to synthesize nano-silicon anode materials from fly ash. The prepared Li-ion battery had a capacitance of 1450.3 mAh·g^−1^ at the current density of 4000 mA·g^−1^, and its reversible capacity remained at 1017.5 mAh·g^−1^ after 100 cycles [109].

Using coal-based solid waste as a low-cost silicon source to prepare porous nano-silicon particles has opened up a new way for the large-scale preparation of Li-ion battery Si-based anode materials and promoted the development of the new energy industry. Additionally, it can effectively reduce the quantity of Si-rich coal-based solid waste and decrease its environmental hazards. However, the volume expansion in Si-based anode materials for Li-ion batteries has not yet been completely solved, resulting in limited cycling performance.

## 4. Conclusions

In summary, coal-gangue-based and fly-ash-based porous functional materials have broad applications in energy and environmental protection, but there are also certain shortcomings. To further achieve large-scale resource utilization of typical coal-based solid waste, the following improvements can be made:

(1) In the field of wastewater treatment, adopt more efficient pretreatment methods (such as pressurized acid leaching, microwave acid leaching, complex acid leaching, etc.) before preparing water treatment materials to reduce heavy metal content in solid waste and focus research on materials with a more stable structure simultaneously, reducing the possibility of secondary pollution.
(2) In the field of waste gas treatment, pre-treat waste gas with means of temperature control and dehumidification before using solid-waste-based gas adsorption materials, minimizing the adverse effects of external factors on the gas adsorption performance of the materials. Some gas adsorption materials that are difficult to regenerate can improve their gas diffusion performance by adding modified substances to form eutectic phases, thereby accelerating the regeneration rate.
(3) In the field of energy regeneration, fully explore the mechanism of various effective components in solid-waste-based catalysts in the catalytic process to achieve efficient and controllable energy regeneration. By loading active metals, regulating the silicon–aluminum ratio, and improving the pore structure, this will improve catalyst energy regeneration efficiency and coking resistance.
(4) In the field of battery manufacturing, the volume expansion problem of Si-based anode materials still requires further exploration of new nanostructures and composite systems to be solved, improving the cycling performance of electrode materials.
(5) In the regeneration process of solid waste, apply technical methods (such as microwave-assisted, ultrasonic-assisted, solvent-free methods, etc.) that can effectively reduce energy consumption and increase productivity in the conversion preparation process of solid waste into functional porous materials, reducing the cost of reusing coal-based solid waste and promoting large-scale resource utilization of typical coal-based solid wastes.

Based on current practical applications, porous materials derived from solid waste still exhibit some deficiencies in the fields of energy regeneration and battery manufacturing. As a result, researching and producing various materials for treating waste gas and wastewater is currently the primary method for reusing typical coal-based solid waste. However, their application in energy will be more promising in the future.

China is a typical country with abundant coal reserves but limited oil and natural gas resources. The development of its industries relies heavily on the extensive mining and combustion of coal. However, the production and accumulation of large amounts of coal-based solid waste over the years have severely harmed the ecological environment. In recent years, with industrial upgrading and the need for ecological protection, the significant shortage of Si-based materials in the fields of energy and environmental protection has provided new approaches for reducing and reusing coal-based solid waste. The large-scale application of various types of coal-gangue-based and fly-ash-based energy and environmental protection porous materials will transform coal-based solid waste from valueless waste into the main force in maintaining ecological stability, promoting the upgrading of energy structures, and achieving national green sustainable development.

## Figures and Tables

**Table 1:** Chemical composition of coal gangue. (Unit: %).

Chemical Composition	SiO_2_	Al_2_O_3_	Fe_2_O_3_	CaO	MgO	Na_2_O	K_2_O	TiO_2_	P_2_O_5_	LOI
Content	30~65	15~40	2~10	1~4	1~3	1~2	1~2	0.5~4.0	0.05~0.3	20~30

**Table 2:** Chemical composition of fly ash. (Unit: %).

Chemical Composition	SiO_2_	Al_2_O_3_	Fe_2_O_3_	CaO	K_2_O	Na_2_O	MgO	SO_3_	LOI
Content	40~60	17~35	2~15	1~10	0.5~4	0.5~4	0.5~2	0.1~2	1~26

**Table 3:** Research status of coal gangue and fly ash in the field of energy regeneration.

Regenerate Objects	Regeneration Effect	Catalytic Material	Preparation Method	Action Mechanism
Waste polyethylene (PE)	Reaction temperature: 700 °C, ratio of feed to catalyst: 20:1, maximum liquid product yield: 78.2%, with total aromatics yield reaching nearly 22%.	Modified fly ash	The coal fly ash was crushed, sieved, and heat-treated at 800 °C to modify [88].	The fly ash catalyst with high specific surface area and high Si/Al ratio provides a large number of highly selective acidic sites, promoting the cracking of plastics and improving the yield of aromatics [89,90].
Waste linear low-density polyethylene (LLDPE)	Catalyst dosage: 15 wt%, LLDPE’s cracking activation energy significantly decreased, and reaction rate and the yield of alicyclic hydrocarbons increased.	Modified fly ash	The coal fly ash was ground to a particle size of less than 150 μm to modify [91].	Solid alkali components such as CaO in fly ash facilitate the formation of carbon anion intermediates, promoting the generation of alicyclic hydrocarbons and accelerating the reaction rate [92]. Transition metal oxide components such as Fe_2_O_3_ in fly ash also assist the plastic cracking process.
Plastic film residue (PFR)	Reaction temperature: 723 K. Use of catalyst inhibits tar and wax formation, reduces cracking temperature, and achieves a 44% product oil yield, with 70% composed of gasoline hydrocarbons.	HX/CFA zeolite	Coal fly ash (CFA) underwent alkali fusion and hydrothermal synthesis to produce NaX zeolite. After acidification, HX/CFA zeolite was obtained [93].	Acidic sites on synthesized X-type zeolites (solid acid) promote the formation of carbocation intermediates in polymer chains, enabling high-quality and efficient cracking of waste plastics [89,90].
Waste wood pellets	A 10 wt% Ni-loaded catalyst and waste wood pellets were steam reformed to obtain a 54.9% gas yield, with an H_2_ production of 7.29 mmol/g.	Ni-ash catalysts	Coal fly ash was impregnated in a Ni(NO_3_)_2_ · 6H_2_O aqueous solution after drying, calcining, reducing it in H_2_ atmosphere to prepare the Ni-ash catalyst [94].	The hydrogenation and dehydrogenation functions of metal Ni, as well as the isomerization, cyclization, and hydrogenation cracking functions of acid components (Al_2_O_3_) in fly ash carrier occur through olefin intermediates during steam reforming. Metals such as Mg and Cu in fly ash also act as catalyst auxiliaries.
Pine sawdust biomass tar	Reaction temperature: 800 °C. The catalyst effectively promoted the decomposition of tar molecules, achieving a conversion rate of 93.5%, and significantly increasing the gas yield.	GC catalysts	The coal gangue (GC) was crushed and sieved, and then calcined at 800 °C for 1 h under N_2_ atmosphere to obtain the GC catalyst [95].	The repeated oxidation and reduction of Fe_2_O_3_ in coal gangue effectively promotes the breaks of C-H bonds and dehydrogenation reactions in catalytic reactions, increasing the yield of H_2_ and CO. Alkali metals in coal gangue further facilitate tar decomposition. The formation of Fe^0^ during the process enhances the activity and lifespan of the catalyst [96,97].
Soybean oil	Reaction temperature: 65 °C, catalyst concentration: 4%, molar ratio of methanol to oil: 12:1, reaction time: 2 h. The conversion rate of soybean oil methyl ester reached 95.5%.	Zeolite-type sodalite	Coal fly ash was added to an alkali solution and supplemented with an aluminate solution to form a gel. The zeolite-type sodalite was formed through hydrothermal crystallization at 100 °C for 24 h using the gel [98].	Solid alkali components (Si-O-Na groups) in zeolite serve as active sites, promoting transesterification to produce biodiesel.
Jatropha curcas oil	Catalyst (40 wt% CaO loaded) dosage: 0.15 wt%, molar ratio of methanol to oil: 12:1, reaction temperature: 60 °C, and reaction time: 1 h. The maximum biodiesel yield was 94.72%.	CaO/CFA catalysts	Coal fly ash was activated by calcination at 400 °C for 5 h, impregnated with a calcium acetate solution and dried, then calcined at 750 °C for 4 h to obtain CaO/CFA catalyst [99].	The appropriate amount of CaO loading improves the catalyst’s pore structure and increases the reaction rate. The introduced CaO (solid alkali) serves as an active site for transesterification, promoting the formation of biodiesel. Si and Al in fly ash act as catalyst carriers to improve catalyst stability.
CO and H_2_	Reaction temperature: 523 K, air pressure: 20 bar, and H_2_/CO = 2. Zeolites carrying more Co particles have higher catalytic activity, resulting in a CO conversion rate of 53.2% and higher selectivity for producing liquid hydrocarbons (C_5+_).	Co/LTA and Co/FAU catalysts	LTA and FAU zeolites synthesized from high-silicon coal fly ash were used as carriers. The Co/LTA and Co/FAU catalysts were obtained by impregnating with Co(NO_3_)_2_ solutions and calcination in H_2_ atmosphere [100].	CO and H_2_ are adsorbed on the surface of metal Co, activated through electron effects and interactions with transition metals, and then dissociated, leading to chain growth and termination to produce various hydrocarbons [101]. Fly-ash-based zeolite carriers plays a role in improving pore structure, enhancing catalyst stability, and increasing the activity of active components during the process.
CO and H_2_	Reaction temperature: 220 °C and air pressure: 30 bar for FT synthesis. After 130 h of intake, the conversion rates of CO and H_2_ were 67% and 73%, respectively. The selectivity for liquid hydrocarbons (C_5+_) was 66.53%. Among C_5+_ hydrocarbons, the contents of gasoline (C_5_–C_11_), diesel (C_12_–C_18_), and high-carbon hydrocarbons (C_19+_) were 33.19%, 27.64%, and 5.7%, respectively.	Co-Fe/SBA-15 catalysts	Coal fly ash was activated by alkali fusion. A small amount of surfactant was added to hydrothermally synthesize mesoporous SBA-15 zeolite. The Co-Fe/SBA-15 catalyst was prepared by impregnating with Co(NO_3_)_2_ and Fe(NO_3_)_3_ solutions and calcining in H_2_ atmosphere [102].	The loading of additional Fe in addition to Co makes the catalyst more advantageous for synthesizing low-carbon hydrocarbons, promoting the generation of gasoline-range products. This catalyst also has a wider CO/H_2_ range and higher poison resistance during F-T synthesis.

## Data Availability

The data presented in this study are available on request from the corresponding author. The data are not publicly available due to technical or time limitations.
